# Designing medical internships to improve recruitment and retention of doctors in rural areas

**DOI:** 10.1080/22423982.2017.1314415

**Published:** 2017-04-18

**Authors:** Margrete Gaski, Birgit Abelsen

**Affiliations:** ^a^ National Centre of Rural Medicine, UiT The Arctic University of Norway, Tromsø, Norway

**Keywords:** Recruitment, remote areas, retention, rural area, workforce, medical internship

## Abstract

**Background**: The medical internship as a way of exposing young doctors to training in a rural context is regarded as a useful tool to recruit and retain doctors in rural areas. Norwegian health authorities tested an arrangement of early sign-up for medical internships in the Finnmark County in Norway.

**Objective**: To report on the effects of the early sign-up for medical internship.

**Design**: This study compares the choice of workplace after internship among physicians who signed up early with those candidates assigned to the raffle model of internship in the study area, and in a comparison area experiencing similar recruitment and retention problems.

**Results**: The proportion of interns who signed up early that still worked as physicians in the study area by April 2014 (29%) was twice as high as among the regular interns (15%) and interns in the comparison area (14%). Among the 59 interns who signed up early still working in the study area in April 2014, 33% had grown up in this area. However, the greatest benefits were for the most densely populated municipalities in the study area.

**Conclusions**: The early sign-up model had a net contribution of proving additional physicians in the study area.

## Introduction

Authorities have used different strategies to recruit and retain physicians in rural and remote areas in many countries [1]. This paper evaluates an early sign-up model for allocating graduates to internships in a rural area [2] for interns who started their internship in primary healthcare between August 2009 and August 2013 in the northernmost county of Norway. The purpose of this article is to evaluate the results of the early sign-up model regarding the recruitment and retention of doctors to a rural area compared to the regular raffle model. The starting point is an outline of theories and research, which is later used to enlighten the results of the early sign-up model compared to the regular raffle model.

### Background variables count and career goals change

The process leading physicians to choose rural practice is complex and variable. The oldest group of theories about career choice is concerned with personal characteristics and searches for “the optimal match” between a job and a person [3,4]. Many studies have demonstrated that the place of origin is a key factor influencing the choice of workplace after medical education and training. Studies from Canada and Australia have concentrated attention on medical graduates with a rural upbringing, who are proven to be more likely to become rural physicians than those with an urban background [5–11]. This means that recruiting graduates with a rural upbringing is regarded as a tool to retain doctors in a rural area. Other studies confirm that medical schools have been successful at graduating doctors who originate from the surrounding area who choose to practice in this area [12–18].

Later theories about career choice are concerned with the fact that career goals will change as a response to claims and opportunities in the environment or as a response to learning experiences [19,20]. Studies demonstrate how rural exposure as a part of medical education leads to a greater likelihood of working rurally after graduation [21,22]. A lesson from this is that the longer the exposure to training in a rural context, the greater the impact on interest in future rural practice and, particularly, the greater the likelihood that doctors make important life decisions in a rural context [22]. The positive association between exposure to rural and remote practice during postgraduate medical training and eventual practice in rural areas in Canada and in Australia has been well documented [23,24]. This means that the medical internship as a way of exposing young doctors to training in a rural context is regarded as a useful tool for recruiting and retaining doctors in rural areas.

### Study context

**Figure 1.**
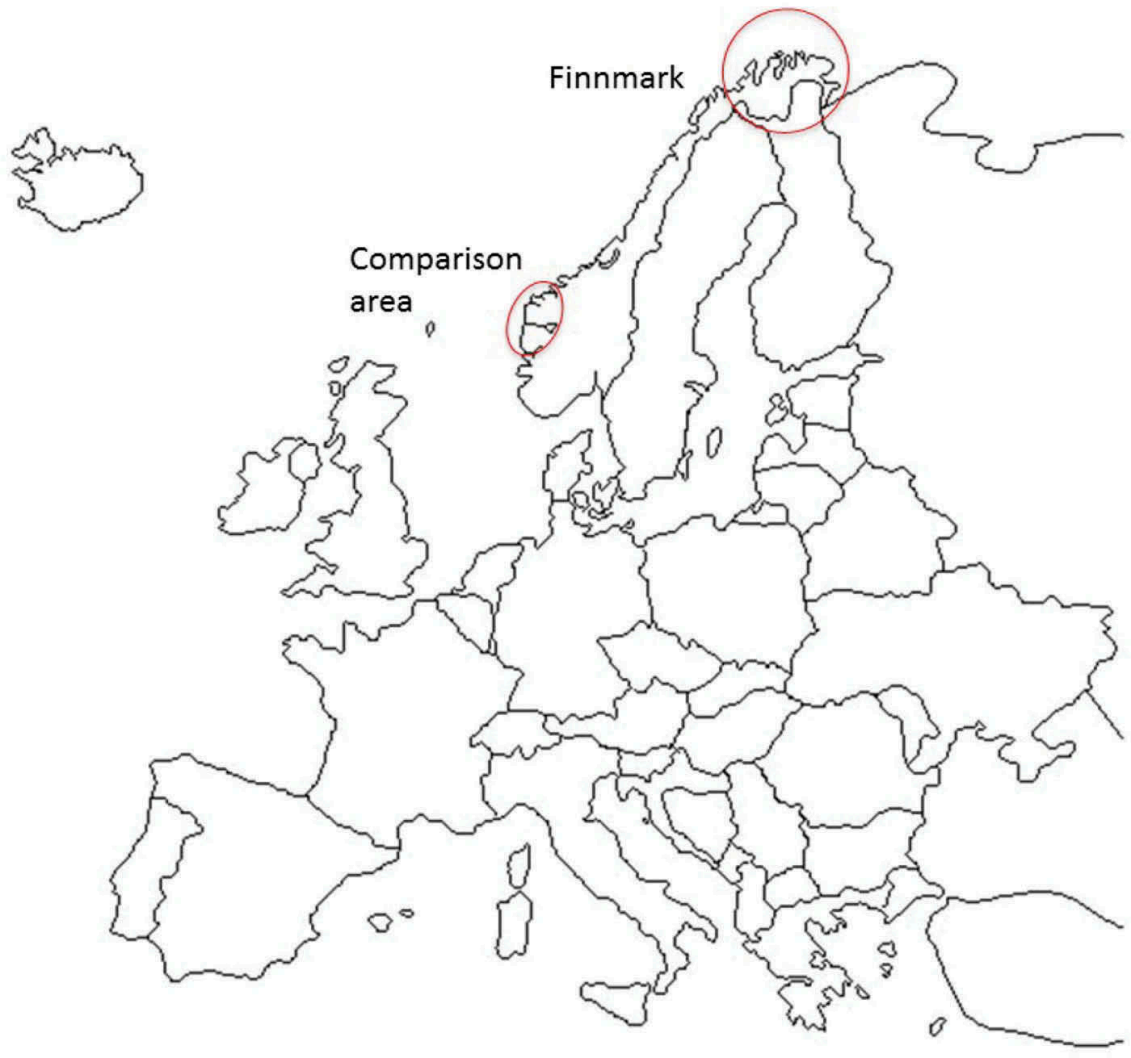
Map including the study area of Finnmark County and the comparison area of Sogn og Fjordane County.

The study area, Finnmark County (Figure 1), is the northernmost county in Norway, covering an area larger than Switzerland, with a population of 75,000 people. There are four major municipalities in Finnmark County, each inhabited by 6000 people or more, and the largest by 20,000 people. Two of these municipalities host small hospitals (with 59 and 95 beds, respectively). There are 15 smaller remote municipalities in Finnmark County (less than 6000 inhabitants).

Medical practice at the primary care level in Finnmark County stands out from other counties because almost all municipalities host one or more general practitioner (GP) beds, where health workers often treat conditions that are normally dealt with in hospitals. The backcloth is long distances and a harsh climate. The total number of physician positons in Finnmark today is 256 (vacant positions included). There are 100 primary care physician posts and 156 positions for physicians in hospitals, psychiatric care included. Recruitment and retention problems have existed more or less since the beginning of the 1960s, both in the local hospitals and in primary care.

The city of Tromsø in the neighbouring county hosts a university hospital and a medical school. The medical school allocate 60% of their seats to students from the surrounding rural and remote area (Finnmark, Troms and Nordland Counties).

The early sign-up model for allocating graduates to internships in this rural area is accompanied by programmes and policies with broader aims to encourage rural recruitment, such as a tailored recruitment project established in 2005 to recruit and retain doctor specialists to the hospitals in the study area, and the Action Zone for Finnmark County (the study area) and the northern part of Troms County, established in 1990, aiming to address high unemployment and generally poor living conditions.

### Internship

Medical internship has been a part of medical training in Norway since 1955. Following medical education (6 years for all students), all candidates have to do postgraduate training (internship) for 1.5 years: graduates spend the first year in hospital, followed by half a year in primary healthcare. Every 6 months, a new group of medical graduates start their internships. After internship, the candidates are ready to start their specialisation, whether they are to be specialised in general practice or any other specialisations. The internship is a possible opportunity for rural exposure.

### Raffle model

Until 2014, interns were distributed by a raffle. All graduates from the medical schools were qualified to participate. The “winner” of this raffle was the first to choose a location for internship placement. The next person chose second, and so on. The most popular internships, and hence the first ones to be chosen, were the urban placements. As the study area is far away from the urban placements, these placements, and especially the placements in the smaller, remote municipalities in the study area, were the last ones to be chosen, and sometimes the internships there remained unoccupied.

### The intervention: the early sign-up model

The previous history leading to the development of the early sign-up model is a special tutorial programme implemented in 1997 by the County Medical Office of Finnmark. Evaluating this programme, Straume and Shaw [2] concluded that medical internship in Finnmark County accompanied by adequate professional and social support might improve recruitment, thereby providing further services in the area. However, the County Medical Office wanted further efforts to recruit and retain more physicians to Finnmark and suggested the early sign-up model. This model gave medical students in their tenth term the opportunity to sign up for internship placement in Finnmark in advance of the regular internship raffle. There were two categories in the early sign-up model: category 1 for the candidates who had geographical affiliations with Finnmark; and category 2 for those who wanted to do their internship in Finnmark, but did not have geographical affiliations with Finnmark. We name both categories as early sign-ups.

The early sign-up logic as a means to improve recruitment and retention of physicians to Finnmark is based on the idea of offering those graduates who are familiar with the area postgraduate training and exposure to medical practice in the area. Early selection would be expected to work because the interns themselves would choose to work in a rural area. We expect them to have a more positive attitude towards working there compared to those who lost the raffle and were forced to go to a rural area. The early sign-up model was in effect from August 2009 until February 2014.

### Research question

The research question was as follows: what are the results of the early sign-up strategy regarding the recruitment and retention of physicians after internships in the study area of Finnmark County compared to similar results of regular internships in Finnmark County and a comparison area?

## Methods

This study compares the choice of workplace following internship among those who signed up early with those assigned to a regular internship placement in the study area (regular interns) and those assigned to a regular internship placement in a comparison area. The comparison area was Sogn og Fjordane County, situated on the west coast in the southern part of Norway, which experienced similar recruitment and retention problems as the study area. The comparison area is populated by 109,000 people, including 26 municipalities, five of them being major municipalities (6000 inhabitants or more), while 20 are remote municipalities (fewer than 6000 inhabitants).

### Data material and analyses

The data material (Table 1) stem from those who started their half-year internship in primary healthcare during the period between August 2009 and August 2013. The Norwegian Registration Authority for Health Personnel has provided lists with names of interns and their hospital internship locations in the two counties during the study period. The County Medical Offices in the two counties involved have provided additional lists with the names of interns and their primary healthcare internship locations in the two counties during the study period.

**Table 1.** Interns included in the study by internship model and area. Interns starting the primary healthcare internship between 1 August 2009 and 1 August 2013. Frequency and percentages are shown (n=388).

	Early sign-up interns in the study area of Finnmark County	Regular interns in the study area of Finnmark County	Regular interns in the comparison area of Sogn og Fjordane County
Total	59	148	181

The mapping of the geographical affiliations of the interns in Finnmark was made in retrospect by asking a network of doctors and staff at the County Medical Office of Finnmark to help highlight who amongst the interns had a geographical affiliation with Finnmark. In this study, geographical affiliation with Finnmark is limited to whether or not the interns themselves were brought up in Finnmark. This limitation is different from the limitation used by the local health authorities, which include interns with a partner who was brought up in Finnmark.

The physicians’ workplaces per April 2014 were mapped. It was registered as to whether they at that time worked in the county where they did their internship or not, and in which sector (hospital or primary healthcare). All physicians working in hospitals were denoted “doctor specialist”, and those working in primary healthcare were denoted “GPs”. The data on workplaces stem from two different sources. The first was human resources personnel in the Finnmark Hospital Trust and the Sogn og Fjordane Hospital Trust, who crosschecked the list of interns in the study period against their lists of employees. The second was the Norwegian Health Economics Administration, who crosschecked the list of interns against a register of all practicing GPs.

The data were mainly analysed by comparing frequencies and percentages. Statistical tests were not performed because population data were analysed.

It is not possible to separate the impacts of the other broader policies for encouraging rural recruitment from the impacts of the early sign-up model because all of these policies work similarly to recruit interns to the study area.

### Ethics approval

The Data Protection Official for Research in Norway approved the study (approval number 38884).

## Results: how effective was the early sign-up strategy?

The material covers 388 candidates, of whom 59 signed up early in the study area, 148 were regular interns in the study area and 181 were regular interns in the comparison area. Women were overrepresented among those who signed up early (71%). The numbers of early sign-up interns vary between 0 and 11 in each 6-month period.

### Higher propensity among early sign-ups to stay

**Figure 2.**
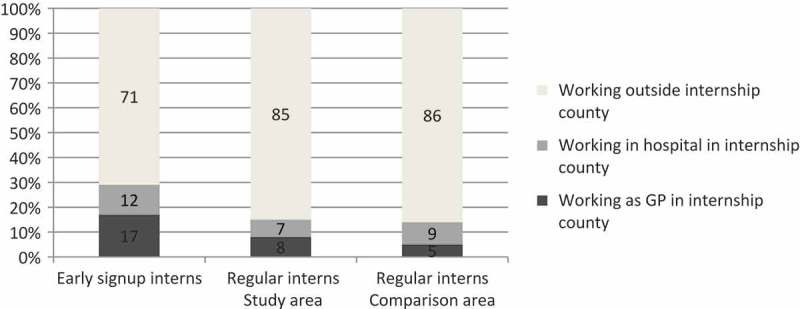
Employment by April 2014. Share of physicians working in or outside their internship county. Former early sign-up interns, regular interns in Finnmark County and regular interns in the comparison area (n=388).

Figure 2 shows that the share of physicians working in Finnmark by April 2014 among those who signed up early was nearly double that compared to the regular interns and slightly more than double that compared to regular interns in the comparison area.

This result is applicable both for physicians working as GPs and for doctor specialists. Among those who signed up early (n=59), 17% were GPs and 12% were doctor specialists in the study area. Among the physicians with regular internships (n=148), 8% were GPs and 7% were doctor specialists in the study area. Among the physicians with regular internships (n=181), 5% were GPs and 9% were doctor specialists in the comparison area.

There is a trend indicating that interns educated in the first third of the study period were less likely to work in the internship county by April 2014 compared to the interns educated during the following years. However, these are small numbers with large variations.

### Early sign-up leads to working in the most densely populated municipalities

Although one could consider the study area (Finnmark County) to be a vulnerable county in relation to recruitment and retention of physicians, there are variations among the municipalities in the county. The early sign-up model does *not* seem to be particularly effective as a means of recruiting GPs to the 15 remote municipalities. Among the 59 interns who signed up early, 44 (75%) chose internships in one of the four most densely populated municipalities.

None of the 59 physicians who had been early sign-up interns worked in any of the 15 remote municipalities in the study area in April 2014. Among the 148 physicians with a regular internship in the study area, four (3%) worked in one of the remote municipalities in the study area at the same time. Among the 181 physicians with a regular internship in the comparison area, three (2%) worked in a remote municipality at the same time.

The internships in the study area during 2009–2014 that were unoccupied were checked. All vacant internships were located to the *remote* municipalities exclusively.

### Upbringing in the study area mattered

**Figure 3.**
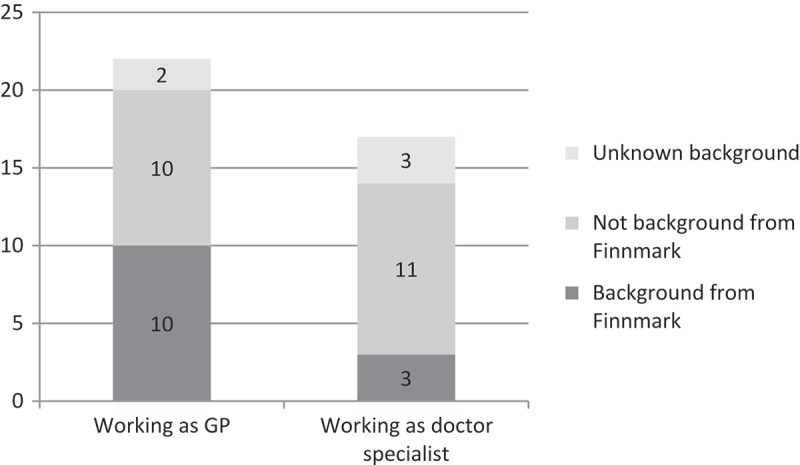
Significance of geographical affiliations with Finnmark among physicians starting the primary healthcare internship between 1 August 2009 and 1 August 2013 working in Finnmark by April 2014 (n=39).

Among the 207 physicians who were interns in the study area (both early sign-ups and regular interns), 39 (19%) were still working in the study area in April 2014. Among them, 13 physicians (33%) had a geographical affiliation with the study area. Among the 22 physicians working as a GP, 10 (45%) had geographical affiliations with the study area, compared to only three among 17 doctor specialists (18%) (Figure 3).

## Discussion

### Discussion of results

The main result is that the share of physicians with an early sign-up internship remaining in the study area was nearly double that compared to physicians with a regular internship, and slightly more than double that compared to physicians with a regular internship in the comparison area.

The main result of the early sign-up strategy is good in the sense that it recruited a much larger amount of interns to work in the study area after their internships. The result is not as good in the sense that it seems to be effective only for the four most densely populated municipalities in the study area and not for the 15 smaller municipalities. This probably reflects that young GPs do not want to work alone or with only one colleague in the smallest and most remote municipalities. Studies have examined the preferences of young GPs for rural locations and pointed to various conditions that need to be present in order to heighten the probability of making a doctor work as a rural GP [25,26]. Among them are a practice size of at least three GPs and good opportunities for professional development. Achieving such working conditions is easier in the most densely populated rural municipalities than in the smallest and most remote municipalities.

When explaining these results, it is reasonable to emphasise the geographical affiliation with the study area among the physicians. The share of GPs with geographical affiliations with the study area still working in the area in April 2014 was considerably higher than the share of doctor specialists in hospitals with geographical affiliations with the study area working there at the same time. This indicates a stronger early sign-up effect among GPs compared to physicians working in hospitals. Professional prestige might play a role in explaining this result, as more prestigious specialities tends to be concentrated in hospitals and urban areas, by default making rural practice less attractive [27]. Several studies have documented the low prestige associated with general practice and family medicine compared to other specialities [28–30]. However, general practice ranks high on lifestyle friendliness-rated specialities [30], and this could be an important factor in play in this particular study setting.

There are some other possible explanations of the results. Conditions in the labour market might be one. The labour market in the study area seems to have been different from the comparison area. In the study area, 11% of the GP positions were vacant or filled by locums (by April 2014). In the comparison area, only 3% of the GP positions were vacant. More positions being vacant made it easier for physicians who wanted to become a GP to start a GP career in the study area. In addition, traditionally, a larger share of doctor specialist positions are vacant both in the study area and in the comparison area compared to central parts of Norway. In this way, the labour market might partly explain why so many of the physicians who did their internships in the study area still worked there in April 2014.

Other parallel recruit and retain measures could also contribute to explaining the results. One measure is a tailored recruitment project established in 2005 to recruit and retain doctor specialists to the hospitals in the study area. This included different measures: scholarships to pay additional costs while spending a compulsory part of the residency in a bigger hospital outside the study area; positions for recruitment; and trainee posts/exchange programmes. Another measure is the Action Zone for Finnmark (the study area) and Northern Troms. The government established this zone in 1990 in response to multiple crises, especially in the fisheries and fishing industry, in order to address high unemployment and generally poor living conditions. This is a mixed bag of measures targeting business, industry and people (including physicians) individually. The current objective is to make this area more attractive as a place to settle, run businesses and work.

The health authorities renewed the Norwegian internship model in 2014. Now, all candidates have to apply for internships in hospitals and municipalities. This system, which replaced the raffle, regards the medical candidates as competitors in the labour market. It is not known what the new market model will bring when it comes to local affiliation as a qualification when engaging interns, and also how this will affect the share of physicians remaining in remote areas after finishing their internships.

### Study limitations

The data material might include some incorrect information regarding registrations of the physicians’ workplaces in April 2014. Our experience is that the national register of all GPs is not up to date and can be delayed by up to 1 year concerning updates on GPs who have quit. The same sort of error could possibly have appeared in the hospitals’ employee lists. These possible errors might be present in the dataset both in the study area and in the comparison area.

The data material from the health authorities, which gives information on the interns, does not include indications of their geographical affiliations with the study area. Our use of a network to map geographical affiliations brings in the possibility of error in the registrations. Similar studies point to methodological challenges in measuring the rural background effect [24] and recommend personal contact as the optimal method for mapping work experience and geographical affiliation. However, using personal contact as a method is more resource intensive and was not applicable to this evaluation.

The early sign-up model included two categories of interns: category 1, which included interns with geographical affiliations with the study area; and category 2, which included others who wanted to be interns in the study area. There are some weaknesses regarding the implementation of the early sign-up model, because there were no lists of which interns were in category 1 and category 2. The result is a mix of three categories among those who signed up early: interns with geographical affiliations with the study area; interns having partners with geographical affiliations with the study area; and interns with no affiliations with the study area (category 2). This makes it more difficult to discuss the impact of giving advantage to interns originating from the study area.

## Conclusions

The early sign-up model had a net contribution of additional physicians to the study area, even though the number of additional physicians recruited through this special arrangement was limited. However, we question whether this arrangement has made an effective contribution to the parts of the study area that are experiencing the most severe challenges with recruiting and retaining physicians.

## References

[CIT0001] Grobler L, Marais BJ, SA Mabunda (2015). Interventions for increasing the proportion of health professionals practicing in rural and other underserved areas (Review). Cochrane database of systematic reviews 2015, Issue 6. Art. No.:CD005314.

[CIT0002] Straume K, Shaw DMP. (2010). Internship at the ends of the earth – a way to recruite physicians?. Rural Remote Health.

[CIT0003] Parsons F (1909). Choosing a vocation.

[CIT0004] Holland JL (1973). Making a vocational choice: a theory of careers.

[CIT0005] Rabinowitz HK (1993). Recruitment, retention, and follow-up of graduates of a program to increase the number of family physicians in rural and underserved areas. New Engl J Med.

[CIT0006] Rabinowitz HK, Diamond JJ, Gayle JA (1998). Alternate career choices of medical students and their eventual specialty choice: a follow-up study. Fam Med.

[CIT0007] Stearns JA, Stearns MA (2000). Graduate medical education for rural physicians: curriculum and retention. J Rural Health.

[CIT0008] Laven G, Wilkinson D (2003). Rural doctors and rural backgrounds: how strong is the evidence? A systematic review. Aust J Rural Health.

[CIT0009] Curran V, Rourke J (2004). The role of medical education in the recruitment and retention of rural physicians. Med Teach.

[CIT0010] Richards HM, Farmer J, Selvaraj S. (2005). Sustaining the rural primary healthcare workforce: survey of healthcare professionals in the Scottish Highlands. Rural Remote Health 5: 365. (Online).

[CIT0011] Hancock C, Steinbach A, Nesbitt TS (2009). Why doctors choose small towns: a developmental model of rural physician recruitment and retention. Soc Sci Med.

[CIT0012] Bertelsen TI (1963). Hvor kommer lægene fra og hvor blir de av? [Where do the physicians come from and where do they go?]. Tidsskr Nor Legeforen.

[CIT0013] Hansen FH (1982). De første Tromsø-medisinerne – hvor er det blitt av dem? [The first Tromsø-physicians - where are they working?]. Tidsskr Nor Legeforen.

[CIT0014] Forsdahl A, Grundnes O, Gamnes J (1988). Hvor blir Tromsø-legene av? [Where do all the Tromsø doctors go?]. Tidsskr Nor Legeforen.

[CIT0015] Magnus JH, Tollan A (1993). Rural doctor recruitment: does medical education in rural districts recruit doctors to rural areas?. Med Educ.

[CIT0016] Inoue K, Hirayama Y, Igarashi M (1997). A medical school for rural areas. Med Educ.

[CIT0017] Alexandersen Ø, Jørgensen E, Østerås J (2004). Medisinerutdanningen i Tromsø – sikrer den legerekrutteringen til Nord-Norge? [Medical school in Tromsø – does it ensure recruitment of physicians to northern Norway?]. Tidsskr Nor Legeforen.

[CIT0018] Aaraas I, Halvorsen P, Chater AB, Rourke J, Couper ID (2014). Developing Rural Medical Schools: History of Tromsø and Northern Norway. WONCA Rural medical education guidebook. World organization of family doctors (WONCA).

[CIT0019] Gintzberg E (1984). Career development: D. Brown & L. Brooks (red.), Career choice and development.

[CIT0020] Bandura A (1977). Social learning theory.

[CIT0021] Playford DE, Evans SF, Atkinson DN (2014). Impact of the Rural Clinical School of Western Australia on work location of medical graduates. Med J Aust.

[CIT0022] Eley DS, Synnott R, Baker PG (2012). A decade of Australian Rural Clinical School graduates – where are they and why?. Rural Remote Health.

[CIT0023] Hogenbirk JC, Mian O, Pong RW (2011). Postgraduate specialty training in northeastern Ontario and subsequent practice location. Rural Remote Health.

[CIT0024] Woolley T, Sen Gupta T, Bellei M (2016). Predictors of remote practice location in the first seven cohorts of James Cook University MBBS graduates. Rural Remote Health.

[CIT0025] Li J, Scott A, McGrail M (2014). Retaining rural doctors: doctors’ preferences for rural medical workforce incentives. Soc Sci Med.

[CIT0026] Holte JH, Kjær T, Abelsen B (2015). The impact of pecuniary and non-pecuniary incentives for attracting young doctors to rural general practice. Soc Sci Med.

[CIT0027] Ono T, Schoenstein M, Buchan J (2014). Geographic imbalances in doctor supply and policy responses. OECD Health Working Papers.

[CIT0028] Aasland OG, Revik JO, Wiers-Jenssen J (2008). Motives for choice of specialty during and after medical school. Tidsskr Nor Legeforen.

[CIT0029] Album D (1991). Sykdommers og medisinske spesialiteters prestisje [The prestige of illness and medical specialties]. Tidsskr Nor Legeforen.

[CIT0030] Creed P, Searle J, Rogers ME (2010). Medical specialty prestige and lifestyle preferences for medical students. Soc Sci Med.

